# Successful Resuscitation of Anaphylactic Shock With Central Nervous System Dysfunction in a Patient on Multiple Antihypertensive Therapies: A Case Report and Literature Review

**DOI:** 10.7759/cureus.101589

**Published:** 2026-01-15

**Authors:** Yojiro Kashimura, Kazushi Hara, Hiroki Shiojima, Motomichi Oki, Koichi Ueno

**Affiliations:** 1 Department of Neurological Endovascular Therapy, Kawasaki Municipal Hospital, Kawasaki, JPN; 2 Department of Emergency and Critical Care Medicine, Kawasaki Municipal Hospital, Kawasaki, JPN

**Keywords:** anaphylactic shock, central nervous system disorder, cns disorder, glucagon, hypertensive patient, iodinated contrast media, multiple antihypertensive therapies, resuscitation, α-blocker, β-blocker

## Abstract

Anaphylaxis in patients taking antihypertensive medications is a life-threatening condition. Early recognition and intervention are crucial to reducing mortality, especially when severe organ dysfunction, such as a central nervous system (CNS) disorder, is present. A 69-year-old male patient with hypertension, who was being treated with amlodipine and carvedilol, was admitted following anaphylactic shock. During contrast-enhanced computed tomography for abdominal pain, he developed sudden hypotension, coma, and a generalized rash. Despite intubation and administration of epinephrine and intravenous glucagon, his hypotension persisted, even after initiating continuous noradrenaline infusion. Following repeated bolus infusions of glucagon, his hemodynamics and consciousness improved. He was extubated after 48 hours and discharged on the fifth day. This case highlights clinical challenges in managing anaphylactic shock in patients using antihypertensive drugs, particularly when presenting with life-threatening CNS precursors. Theoretically, epinephrine increases blood pressure via α1- and β1-receptor stimulation; however, concurrent use of β-blockers can impair these responses. In this patient’s etiology, glucagon was essential to bypass the β-adrenergic receptors. For patients on α-blockers, vasopressin may serve as a second-line treatment for noradrenaline-resistant shock by acting on vasopressin receptors. Extracorporeal membrane oxygenation should be considered if pharmacological management fails. Furthermore, although this patient was resuscitated with the current protocol, literature suggests that intravenous epinephrine may be necessary if intramuscular administration is ineffective in severe cases. In conclusion, we successfully managed a complex case of anaphylactic shock with CNS dysfunction. Given the lack of robust evidence for managing such refractory cases, further accumulation of case reports or observational studies is warranted.

## Introduction

Anaphylaxis is a well-recognized complication of contrast-enhanced computed tomography (CECT) scans. The most severe form “anaphylactic shock” is characterized by hypotension and carries high risk of mortality, particularly when accompanied by severe organ dysfunction, such as hypoxic hepatitis (“shock liver”), acute kidney injury (“shock kidney”), impaired consciousness (“shock brain” and central nervous system (CNS) dysfunction induced by cerebral hypoperfusion) [1]. Despite its clinical significance, several aspects of managing severe anaphylactic shock remain poorly understood [1].

Iodinated contrast media (ICM) were introduced to clinical practice in the early 20th century. Although their use has increased since the development of low-toxicity agents in the 1950s, adverse events remain a clinical concern. The exact mechanism of ICM-induced anaphylaxis has remained a subject of debate for decades; it is generally classified into IgE-mediated and non-IgE-mediated pathways. Furthermore, anaphylaxis is triggered by complement activation-related pseudo-allergy (CARPA) as well [1-5].

The management of anaphylactic shock could be particularly challenging in patients taking medications that affect cardiovascular dynamics. Previous reports have suggested that patients receiving β-blockers can manifest refractory shock, for which glucagon may be supportive. However, due to the paucity of such cases, the evidence for glucagon therapy remains limited [5-7]. Additionally, the patient presented in this case report was taking amlodipine, a calcium channel blocker (CCB). Previous literature has highlighted the potential for CCBs to exacerbate the severity of anaphylaxis [8]. Furthermore, this patient was on α-blocker therapy as well. Notably, there have been limited reports of anaphylactic shock in patients taking α-blockers [9].

In summary, the mechanism and optimal management of the most serious form of anaphylaxis, anaphylactic shock with severe organ dysfunction, in patients on multiple antihypertensive medications remains unclear. Therefore, we here describe and discuss a case of an ICM-induced anaphylactic shock complicated by a CNS disorder.

## Case presentation

**Figure 1:**
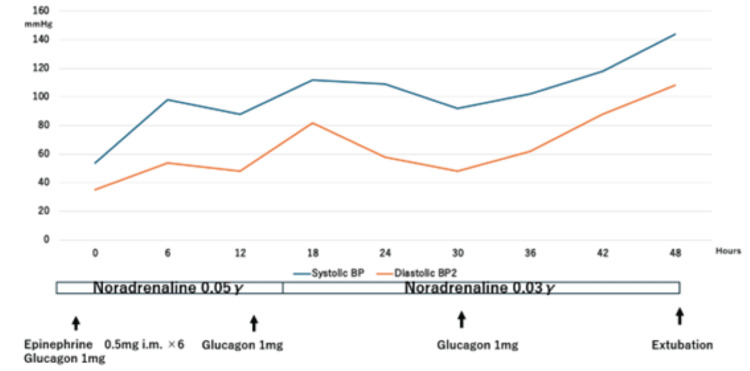
Timeline of blood pressure management in the patient This figure shows the patient's blood pressure over time. It was sustained by the vasopressor and bolus glucagon infusions. He was extubated 48 hours after iodinated contrast media.

A 69-year-old male patient with a history of hypertension and scoliosis was admitted to our hospital following anaphylactic shock. His medications included amlodipine (5 mg/day) and carvedilol (2.5 mg/day), with no known allergies. During CECT for abdominal pain at another hospital, he developed sudden hypotension (blood pressure (BP) 54/35 mmHg; normal values: 130-90/80-60 mmHg), dyspnea, and loss of consciousness (Glasgow Coma Scale (GCS) E1V1M3) (Figure 1).

He was immediately intubated and received six doses of intramuscular (IM) epinephrine (0.5 mg each), followed by intravenous (IV) glucagon (1 mg), methylprednisolone (80 mg), and diazepam (10 mg). Despite the initiation of continuous IV norepinephrine at 0.05 μg/kg/min, hypotension persisted. Following an additional bolus of 1 mg IV glucagon, his BP improved to 98/56 mmHg and his GCS score rose to E1VtM4. He was then transferred to our intensive care unit.

Upon arrival at our emergency intensive care unit, his vital signs were GCS E1VtM5 (normal values: E4V5M6); heart rate 96 beats/min (normal values: 50-110 beats/min); BP 108/57 mmHg; respiratory rate 15 breaths/min (normal values: 7-25 breaths/min); and body temperature 36.8°C (normal values: 35.1-36.9°C). Physical examination revealed a generalized rash on his face, chest, and extremities. Chest auscultation revealed bilateral inspiratory wheezing. Laboratory findings included a white blood cell count of 14,800/μL and a lactate level of 7.5 mmol/L (Table 1).

**Table 1:** Initial laboratory data WBC: white blood cells; Neu: neutrophil; Eos: eosinophil; Lym: lymphocyte; Mono: monocyte; Baso: basophil; RBC: red blood cells; Hb: hemoglobin; APTT: activated partial thromboplastin time.

Test	Result	Reference range
WBC (/μL×10^3^)	14800*	3500-9000
Neu (%)	81.6*	38-74
Eos (%)	0.2	0-10
Lym (%)	10.6*	16.5-49.5
Mono (%)	7.5	5.0-10.0
Baso (%)	0.1	0-2
RBC (/μL×10^3^)	490	350-555
Hb (g/dL)	13.7	13-16
Platlets (/μL×10^4^)	30.1	15-40
APTT (sec)	20.0*	24.0-39.0
Fibrinogen (mg/dL)	274.9	200-400
Total protein (g/dL)	5.2*	6.2-7.5
Albumin (g/dL)	3.2	3.0-3.6
Total bilirubin (mg/dL)	0.5	0.4-1.5
Aspartate aminotransferase (U/L)	28	13-30
Alanine transaminase (U/L)	43*	13-23
Blood urea nitrogen (U/L)	16	7.5-17.5
Creatinine (mg/dL)	0.94	0.5-1.8
Sodium (mmol/L)	150*	136-147
Chlorine (mmol/L)	112*	102-108
Potassium (mmol/L)	3.1*	3.4-4.7
Calcium (mg/dL)	7.2*	8.2-10.0
Phosphate (mg/dL)	2.3	2.5-4.5
Magnesium (mg/dL)	2.4	1.5-2.2
Glucose (mg/dL)	97	73-109
Lactate (mmol/L)	7.5*	0.5-2.0
C-reactive protein (mg/dL)	0.05*	＜0.3

Shortly after admission, the patient experienced a recurrent seizure while his BP was 98/54 mmHg. Following the administration of IV diazepam (10 mg) and glucagon (1 mg), the seizure ceased, and his BP increased to 118/72 mmHg. Fifteen hours after the initial ICM infusion, his BP dropped again to 88/48 mmHg. A bolus of IV glucagon (1 mg) successfully increased his BP to 126/78 mmHg. Continuous IV norepinephrine was tapered to 0.03 μg/kg/min 23 hours post-ICM infusion, at which point his GCS was E3VtM5.

Forty-five hours after the ICM infusion, his BP stabilized at 122/82 mmHg, and norepinephrine was discontinued. As his BP remained stable at 124/86 mmHg and his GCS score improved to E4VtM6, he was extubated 48 hours after ICM administration. No recurrence of symptoms occurred post-extubation, and the patient was discharged on the fifth day of admission. Subsequent skin tests performed in the dermatology department confirmed the diagnosis of anaphylaxis to ICM.

## Discussion

This case study highlights the uncommon and novel aspects of anaphylactic shock presenting with CNS dysfunction and discusses treatment strategies based on a literature review.

First, the patient experienced anaphylactic shock accompanied by coma induced by an ICM. Although anaphylaxis is relatively common in clinical practice, its precise mechanisms remain elusive. Anaphylactic shock accompanied by severe organ dysfunction, particularly CNS involvement, is regarded as a precursor to high mortality [1]. Unlike other organ dysfunctions such as hypoxic hepatitis (“shock liver”) or acute kidney injury (“shock kidney”), impaired consciousness (“shock brain”) can be evaluated immediately at the bedside without awaiting laboratory or radiological results. Therefore, clinicians must recognize its diagnostic utility and the necessity for prompt intervention.

However, there are currently insufficient reports to establish optimal management protocols [1]; thus, this case serves as a valuable contribution to the clinical literature. Although the positive skin test in this case suggests an IgE-mediated mechanism, the classification of ICM-induced anaphylaxis remains a matter of controversy [1-6]. While this report adds to the cumulative evidence for ICM-induced anaphylactic shock, further studies are required to clarify its classification in real-world clinical settings.

Furthermore, there is no established management strategy for refractory anaphylaxis presenting with severe CNS dysfunction, particularly in patients taking multiple antihypertensive medications that adversely affect cardiovascular dynamics. Theoretically, epinephrine increases BP by stimulating α1- and β1-receptors; α1-receptors mediate peripheral vasoconstriction, while β1-receptors enhance positive cardiac inotropic effects. Vasopressin also induces peripheral vasoconstriction and is utilized as a second-line treatment for catecholamine-resistant shock [9-11].

According to recent studies, the indication for glucagon in anaphylactic shock for patients on β-blocker therapy remains controversial. Although a previous report suggested that these patients might not require escalated doses of epinephrine [12], Murakami et al. recently reported a case where glucagon was successfully used in a patient on β-blockers [13]. They emphasized that glucagon was highly effective and warranted further clinical evaluation. In the present case, glucagon was clinically effective; therefore, we also recommend its further evaluation in similar refractory cases. 

In addition, this patient was also taking amlodipine. Although the patient was successfully resuscitated with repeated IM epinephrine and continuous IV noradrenaline, previous reports suggest the necessity of alternative treatment options in similar cases. Hammad et al. reported a case of refractory anaphylactic shock in a patient taking amlodipine, where BP could not be managed by IM epinephrine alone, and required administration of IV epinephrine. Since the effects of vasopressors can be attenuated by the presence of CCBs, maintaining circulatory stability with IM epinephrine may be challenging. Therefore, IV epinephrine should be considered if signs of serious organ dysfunction persist despite repeated IM injections [8]. 

Furthermore, carvedilol possesses α-blocking activity, which contributed to the difficulty in maintaining the patient’s BP. Although reports of anaphylactic shock in patients taking α-blockers are limited, Nakano et al. reported a case of anaphylaxis in a patient treated with risperidone, which also exerts α-blocking effects [9]. They suggested that the chronic use of α-blockers is a significant risk factor for epinephrine-resistant anaphylactic shock. In such cases, vasopressin may be theoretically required, as it induces peripheral vasoconstriction via vasopressin receptors rather than adrenoceptors [8,11]. Extracorporeal membrane oxygenation (ECMO) should be considered when circulatory dynamics cannot be stabilized through pharmacological management [8,11].

## Conclusions

In conclusion, this report describes a case of anaphylactic shock induced by ICM accompanied by CNS dysfunction, a known predictor of high mortality, in a patient taking multiple antihypertensive medications. Despite the severity of the condition, the patient was successfully treated. We have summarized the treatment strategies for anaphylactic shock presenting with circulatory dysfunction. However, given the scarcity of similar cases, further evaluation through additional case reports or observational studies is recommended to establish robust evidence for managing the most severe forms of anaphylactic shock.
